# Differential analysis of clinical outcomes in cerebral infarction associated with REM-OSA and NREM-OSA: a retrospective database study

**DOI:** 10.3389/fneur.2025.1607963

**Published:** 2025-07-15

**Authors:** Liwen Xu, Wenyi Yu, Shutong Sun, Yixi Zheng, Tianyu Jing, Gang Xu, Tieyu Tang, Cheng Chu

**Affiliations:** ^1^Department of Neurology, The Affiliated Hospital of Yangzhou University, Yangzhou University, Yangzhou, China; ^2^School of Nursing and School of Public Health, Yangzhou University, Yangzhou, China

**Keywords:** cerebral infarction, rapid eye movement phase, obstructive sleep apnea, hypoxia, inflammation, basal ganglia, prognosis

## Abstract

**Background:**

Obstructive sleep apnea (OSA) is one of the factors that affect the prognosis of cerebral infarction. Rapid eye movement-related obstructive sleep apnea (REM-OSA) has been confirmed as an important clinical subtype of OSA, yet it is frequently overlooked in clinical practice. REM-OSA is an important but underrecognized clinical issue in the study of improving the prognosis of cerebral infarction.

**Objective:**

To investigate the relationships among REM-OSA and cerebral infarction clinical prognosis.

**Methods:**

In this retrospective cohort study, 318 cerebral infarction patients with OSA (AHI ≥ 5) were enrolled from February 2022 to January 2025 at the Department of Neurology, Affiliated Hospital of Yangzhou University. Participants were stratified into REM-OSA (*n* = 71) and NREM-OSA (*n* = 247) groups using stringent criteria (AHI_REM_/AHI_NREM_ ≥ 2, REM duration ≥30 min). Data included polysomnography, neurological assessments (NIHSS, MRS), inflammatory markers (WBC, hs-CRP), and neuroimaging. Statistical analyses comprised logistic regression and Pearson correlation tests.

**Results:**

Compared to NREM-OSA, REM-OSA patients exhibited: (1) Poorer prognosis: Higher 3-month mRS scores (OR = 1.543, *p* = 0.032), independent of total AHI. (2) Enhanced inflammation: Elevated WBC (7.45 vs. 6.50 × 10^9^/L, *p* = 0.011) and hs-CRP (3.95vs.1.16 mg/L, *p* < 0.001), correlating with AHI_REM_ (*r* = 0.234–0.268, *p* < 0.001). (3) Unique neuroanatomical vulnerability: Higher basal ganglia infarction prevalence (83.1% vs. 64.8%, *p* = 0.003; OR = 2.359). (4) Severe REM-specific hypoxia: Lower minimum SpO₂ (81.62% vs. 84.31%, *p* < 0.001) and prolonged apneas. (5) Sleep architecture disruption: Reduced sleep efficiency and prolonged latency (PSQI: 10.18 vs. 8.52, *p* = 0.004). (6) Age inversely correlated with REM-OSA severity (*r* = −0.154, *p* = 0.020).

**Conclusion:**

REM-OSA is independently associated with poorer prognosis in cerebral infarction patients. Potential explanatory mechanisms include REM-specific hypoxia, systemic inflammation, and basal ganglia vulnerability.

## 1 Introduction

Obstructive sleep apnea (OSA) is a sleep disorder characterized by partial or complete collapse of the upper airway during sleep, leading to fragmented sleep and intermittent hypoxemia. This condition is accompanied by a variety of comorbidities and poses a serious risk to public health (1).

Rapid eye movement-dominant obstructive sleep apnea (REM-OSA) is a distinct clinical phenotype of OSA (2) that is characterized by apnea and hypopnea events that primarily occur during the REM sleep period and is often considered more severe due to the reduced activation of dystonia during this period (3). The undefined nature of REM-OSA has led to variations in its prevalence, with studies suggesting that it accounts for approximately 10 to 36% of the OSA population (4, 5). It has been shown to be associated with the cardiovascular, endocrine, and neurological systems (6) and has detrimental effects on people and society.

Cerebral infarction is a serious clinical condition with a poor prognosis and high mortality. OSA has been identified as an independent risk factor for cerebral infarction (7), and the incidence of OSA after stroke can reach 80% (8). Recent studies have shown that OSA affects cerebral haemodynamics, brain injury, and autonomic dysfunction to varying degrees (9), and it may also be an important predictor of serious adverse outcomes after stroke (10). However, studies on REM-OSA and its clinical outcomes in cerebral infarction patients are lacking. REM-OSA is characterized by a lower average apnea hypopnea index (AHI), making it easy to neglect in clinical practice. Therefore, to investigate the prognosis of cerebral infarction, whether REM-OSA should be considered an important OSA subtype requiring active treatment needs to be further studied.

This study focused on the laboratory indices of REM-OSA patients after cerebral infarction, the location of cerebral infarction, polysomnographic data, and neurological function. The aim of this study was to explore the differences between REM-OSA and NREM-OSA in patients with cerebral infarction, and to provide a new approach for improving the clinical prognosis of cerebral infarction patients.

## 2 Materials and methods

### 2.1 Participants

In this retrospective cohort study, we included clinical data from patients with a diagnosis of cerebral infarction at the Department of Neurology, the Affiliated Hospital of Yangzhou University, from February 2022 to January 2025, as well as objective and subjective sleep data at the sleep center, with follow-up completed 3 months after discharge, and we used neurologic scores representing the primary outcome. The inclusion criteria for patients were as follows: (1) aged ≥18 years; (2) met the diagnostic criteria for stroke in the Chinese Guidelines for the Diagnosis and Treatment of Acute Ischaemic Stroke 2018 (11) and had ischaemic stroke confirmed by cranial CT or magnetic resonance (MRI) imaging; (3) completed a full night of polysomnography (PSG) with an AHI of ≥5 events per hour, along with complete sleep data. The exclusion criteria were as follows: (1) known comorbidity with OSA and previous treatment with positive pressure ventilation; (2) total REM sleep time <30 min as monitored by PSG; (3) cognitive dysfunction; (4) previous history of pharyngeal cavity reconstruction surgery; (5) incomplete sleep monitoring or missing data. The study was approved by the Ethics Committee of Yangzhou University (Ethics 2023-YKL09), and patients signed an informed consent form before entering the study.

### 2.2 Information collection

#### 2.2.1 General information

Baseline information, such as age, sex, BMI, smoking and drinking history, and previous medical history were included in my analysis. Blood-related indices, such as white blood cells, neutrophils, high-sensitivity C-reactive protein triglycerides (hs-CRP), cholesterol, homocysteine (hcy), blood creatinine, and glycated haemoglobin, were also included. Additionally, imaging indices, such as CT and MR images of the head, were included in my research.

#### 2.2.2 Subjective sleepiness scale information

The Epworth Sleepiness Scale (ESS) is the most commonly used questionnaire for assessing daytime sleepiness in patients with sleep disorders (12). The ESS consists of eight questions that assess the likelihood of dozing off in eight different situations over the past month. The scale consists of 24 points, and a score of >6 indicates drowsiness, >11 indicates excessive drowsiness, and >16 suggests dangerous drowsiness.

The Pittsburgh Sleep Quality Index (PSQI) is utilized to evaluate the sleep quality of patients, and it has demonstrated good reliability and validity in assessing the sleep quality of patients with psychiatric and sleep disorders as well as those with various somatic disorders (13). The scale comprises nine questions, and the scoring system encompasses seven components: sleep quality, sleep onset latency, sleep duration, sleep efficiency, sleep disturbances, use of sleep medications, and daytime dysfunction. The total score ranges from 0 to 21, with higher scores indicating poorer sleep quality.

#### 2.2.3 Neurologic function assessment

The National Institutes of Health Stroke Scale (NIHSS) is a comprehensive and objective semiquantitative assessment tool for stroke severity. It has prognostic value (14) and consists of a total of 42 points, with higher scores indicating more severe neurological damage. In this study, it was used as a prognostic indicator to evaluate stroke severity upon admission and discharge.

The Modified Rankin Scale (MRS) is utilized to assess neurological function recovery in stroke patients (15), with higher scores indicating poorer neurological recovery. The short-term functional outcome of the patients was assessed 3 months after discharge from the hospital during the follow-up.

The above NIHSS score was assessed at the time of onset, whereas the MRS score was obtained 3 months after discharge as a prognostic indicator. Higher NIHSS and mRS scores indicate a worse prognosis.

### 2.3 Polysomnography

#### 2.3.1 Sleep monitoring

All patients underwent polysomnographic sleep apnea monitoring at the Sleep Testing Center of The Affiliated within 3 days of admission Hospital of Yangzhou University. The monitoring included EEG, electrooculogram, mandibular electromyogram, ECG, respiration (using a snoring sensor, thermography, and airflow monitoring), thoracic and abdominal movements, blood oxygen level monitoring, leg movements, and sleep position tracking. On the following day, two professionally trained readers analysed the sleep charts. If the results were inconsistent, a third reader made the decision. The PSG monitoring process followed the standards outlined in the American Academy of Sleep Medicine Manual for the Interpretation of Sleep and Its Associated Events (16). The analysis included indicators such as REM latency, REM%, REM duration, AHI_REM_, AHI_NREM_, an oxygen desaturation index (ODI) ≥ 3, the arousal index, and the respiratory event-related arousal index.

#### 2.3.2 Diagnostic and grouping criteria

Given the lack of standardization in the diagnosis of REM-OSA and NREM-OSA, we combined Jose Haba-Rubio et al. (17) previous research with practical considerations to define the REM-OSA group as AHI_REM_/AHI_NREM_ ≥ 2, with at least 30 min of REM sleep, and the NREM-OSA group as AHI_REM_/AHI_NREM_ < 2. All patients received formal continuous positive airway pressure titration. Sleep specialists nurses provided them with health education about CPAP, and followed up and urged them.

### 2.4 Statistical methods

Our study was conducted and the data were analysed according to the Strengthening the Reporting of Observational Studies in Epidemiology (STROBE) guidelines (18). An Excel database was created, and the data were analysed using the SPSS 26.0 software package (IBM Corp., Armonk, USA). Normally distributed data are expressed as $\bar{X}\pm S$ , whereas non-normally distributed data are expressed as the median (quartile) [M(P25, P75)]. For between-group comparisons, the independent samples *t*-test or rank sum test were used. Count data are expressed as [n(%)]. Measurements were tested using the chi-square (χ^2^) test, and the Spearman rank correlation test was used to analyse the relationship between the AHI_REM_ and other count data. Binary logistic regression was utilized to identify the pertinent variables of REM-OSA and to analyse the causal relationship between REM-OSA and prognostic indicators of cerebral infarction. All the statistical tests were two-sided, and 95% confidence intervals (95% CIs) and odds ratios (ORs) were reported. Differences were considered statistically significant at *p* < 0.05.

## 3 Results

### 3.1 Comparison of baseline data between the two groups of patients

**Figure 1:**
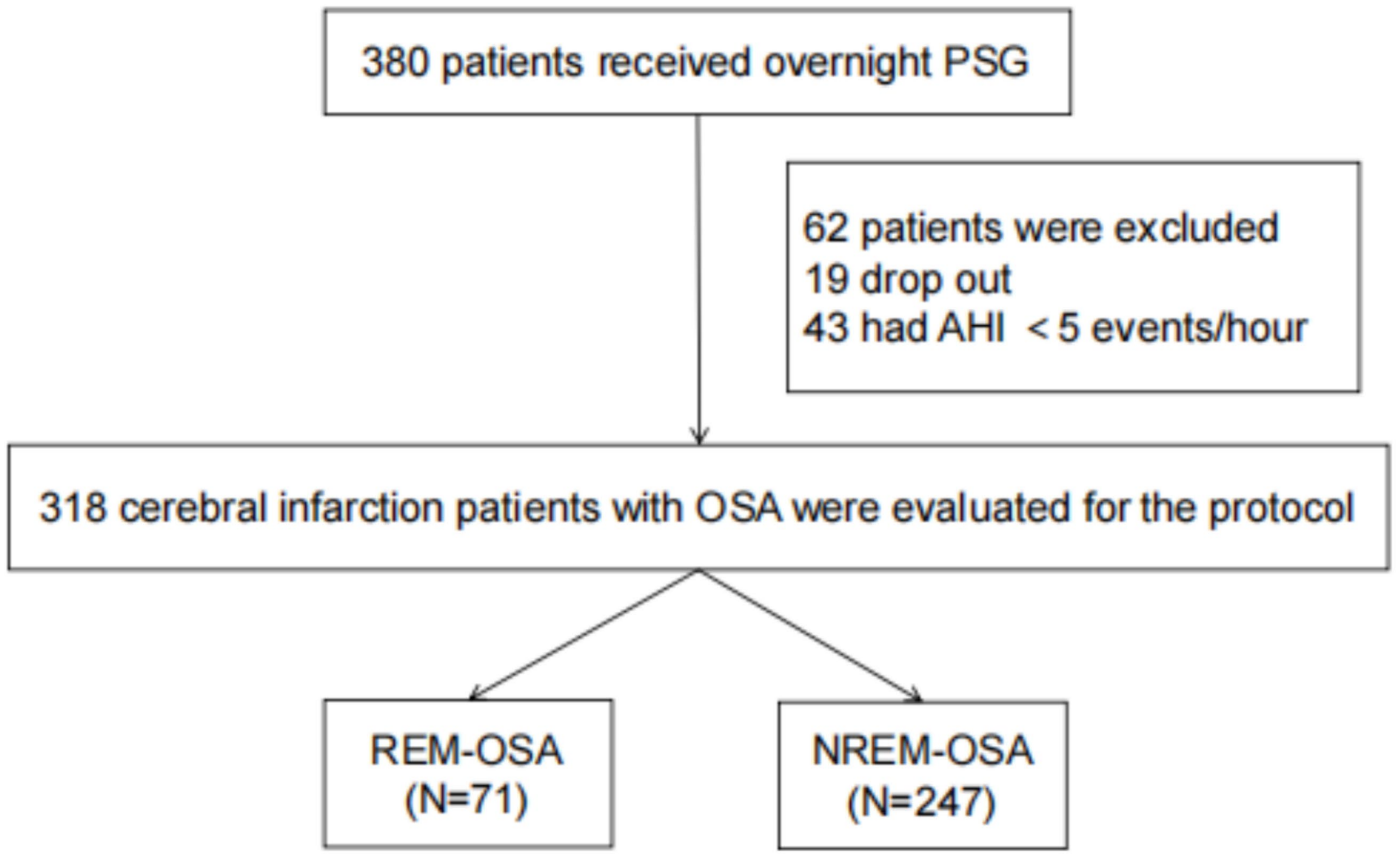
Consort flow chart of the study sample. PSG, polysomnography; AHI, apnea-hypopnea index; OSA, obstructive sleep apnea; REM, rapid eye movements; NREM, non-rapid eye movements.

**Figure 2:**
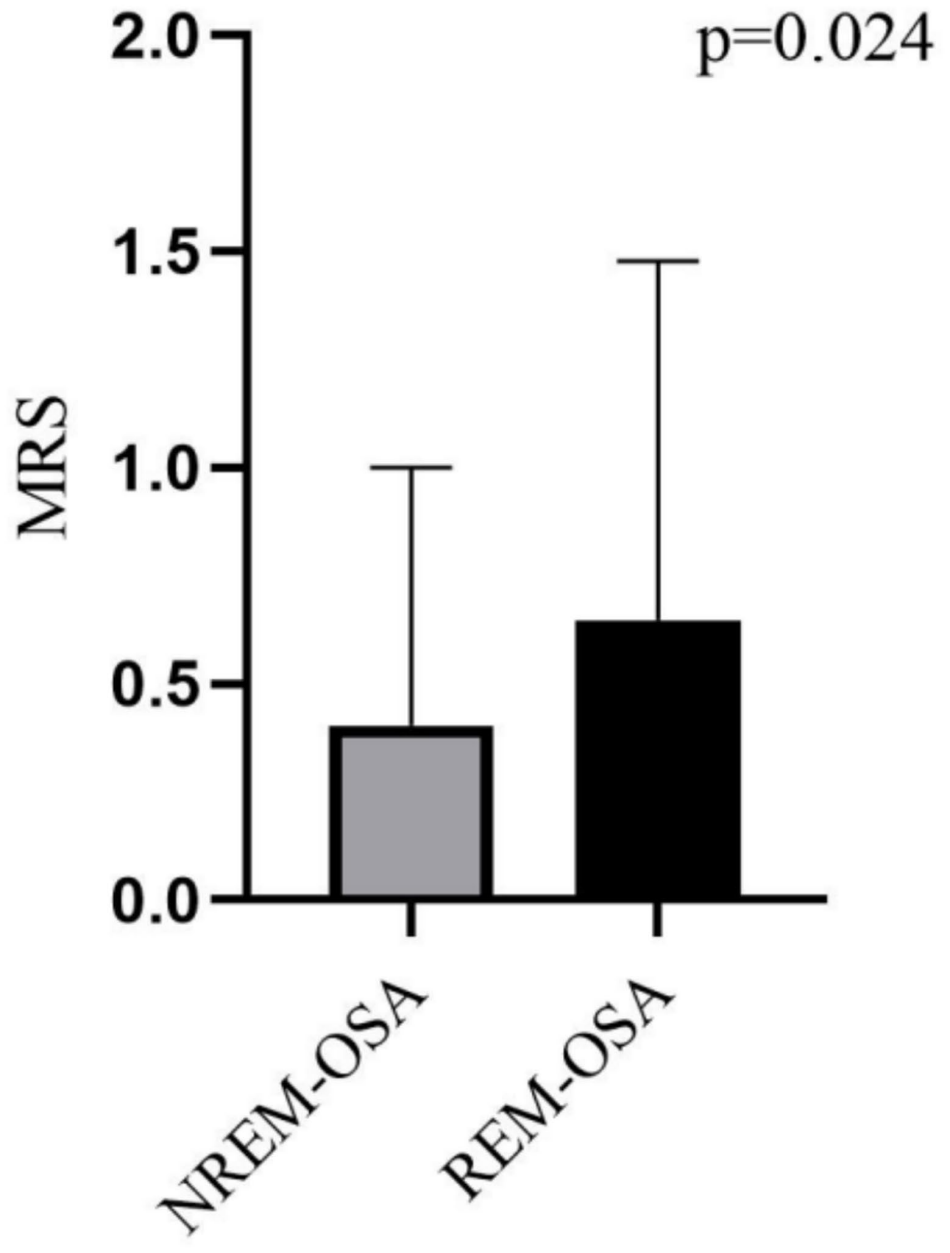
Differences in MRS between NREM-OSA and REM-OSA.

As shown in Figure 1, 380 patients in the entire cerebral infarction cohort underwent all-night polysomnography. Among them, 318 (83.68%) cerebral infarction patients were diagnosed with OSA. This diagnosis excluded 19 patients who withdrew due to intolerance and 43 patients with an AHI of less than 5 events/h. The patients with OSA were categorized into the REM-OSA group, consisting of 71 patients (22.33%), and the NREM-OSA group, consisting of 247 patients (77.67%). The baseline data for the two groups of patients were compared, as shown in Table 1. The analysis revealed a significant difference in MRS Scores between the two groups at 3 months after discharge (*p* < 0.05), as shown in Figure 2.

**Table 1:** Baseline demographic and clinical characteristics of the study population.

Variables	NREM-OSA	REM-OSA	t/z/χ^2^	*p*
Age, years	65.57 ± 11.292	65.18 ± 9.471	0.261	0.794
Female sex, %	68.3%	61.2%	0.575	0.061
BMI, kg/m^2^	25.599 ± 3.603	25.942 ± 3.259	−0.722	0.471
Smoking, %	25.1%	19.7%	0.879	0.349
Drinking, %	21.9%	18.8%	0.775	0.102
Hypertension, %	78.1%	76.1%	0.138	0.711
Diabetes mellitus, %	37.2%	39.4%	0.113	0.737
History of coronary heart disease, %	10.5%	8.5%	0.263	0.608
NIHSS (before)	0.00(0.00–1.00)	0.00(0.00–1.00)	−1.513	0.134
MRS (before)	0.00(0.00–1.00)	0.00(0.00–1.00)	−1.650	0.102
MRS (after)	0.00(0.00–1.00)	0.00(0.00–1.00)	−2.303	0.024

BMI, body mass index. NIHSS, National Institutes of Health Stroke Scale. MRS, Modified Rankin Scale.

### 3.2 Comparison of laboratory indicators

As shown in Table 2, white blood cell (WBC), neutrophil (NE) counts and high-sensitivity C-reactive protein (hs-CRP) were significantly greater in the REM-OSA group than in the NREM-OSA group (P < 0.05). Additionally, no statistically significant differences (*p* > 0.05) were detected in the triglyceride, cholesterol, high-density lipoprotein, low-density lipoprotein, homocysteine, blood creatinine, or glycosylated haemoglobin levels between the two groups.

**Table 2:** Comparison of laboratory indices of the study participants.

Variables	NREM-OSA	REM-OSA	t/z	*p*
WBC, 10^9^/L	6.496 ± 1.748	7.448 ± 2.929	−2.617	0.011
NE,10^9^/L	4.511 ± 1.737	4.500(3.590,6.000)	−2.148	0.000
hs-CRP, mg/L	1.16(0.500,2.710)	3.950(1.470,5.900)	−4.651	0.000
TG, mmol/L	1.440(1.030,2.070)	1.530(1.150,2.230)	−0.340	0.734
TC, mmol/L	4.060(3.350,4.750)	4.330(3.680,5.330)	−1.718	0.087
LDL-C, mmol/L	2.602 ± 0.890	2.797 ± 0.882	−1.624	0.105
HDL, mmol/L	1.187 ± 0.334	1.208 ± 0.482	−0.430	0.667
Hcy, μmol/L	10.300(6.500,13.410)	9.700(7.700,13.550)	0.212	0.833
SCR, μmol/L	75.038 ± 37.961	68.355 ± 22.062	1.415	0.158
HbA1c, %	6.671 ± 1.644	6.501 ± 1.349	0.794	0.428

WBC, white blood cell; NE, neutrophilic granulocyte; hs-CRP, high-sensitivity C-reactive protein; TG, triglyceride; TC, total cholesterol; LDL-C, low density lipoprotein cholesterol; HDL, high density lipoprotein Hcy, homocysteine; SCR, serum creatinine; HbA1c, glycosylated haemoglobin.

### 3.3 Comparison of infarction single/bilateral, infarction single/multiple, number of SCIs, and infarction localization in the two groups

As shown in Table 3, the REM-OSA group exhibited a significantly greater percentage of cerebral infarction sites in the basal ganglia than did the NREM-OSA group (*p* = 0.003). There was no significant difference between the two groups in terms of infarcts mono/bilateral, infarcts single/multiple, number of infarcts, or infarcts located in the subcortical layer, cortex, brainstem, or cerebellum (*p* > 0.05).

**Table 3:** Comparison of the type, laterality, multiplicity, number of SCIs, and location of the study participants.

Variables	NREM-OSA	REM-OSA	χ^2^	P
Laterality				
Unilateral, %	8.1	9.9	0.220	0.639
Bilateral, %	91.9	90.1		
Multiplicity				
Isolated,%	10.5	9.9	0.026	0.871
Multiple, %	89.5	90.1		
Number of SCI				
≤4, %	60.7	47.9	3.730	0.053
>4, %	39.3	52.1		
Localization				
Basal ganglia, %	64.8	83.1	8.634	0.003
Subcortical, %	90.3	93.0	0.476	0.490
Cortex, %	90.7	87.3	0.690	0.406
Brainstem, %	15.0	23.9	3.143	0.076
Cerebellum, %	5.7	9.9	0.965	0.326

SCI, silent cerebral infarct.

### 3.4 Comparison of drowsiness and sleep quality differences between the two groups of patients

As shown in Table 4, there was no significant difference in the degree of sleepiness between the two groups (*p* = 0.607). The quality of sleep in the REM-OSA group, as assessed using the PSQI, was poorer than that in the NREM-OSA group (*p* = 0.004). Furthermore, the time taken to fall asleep, as indicated by the subdimensions of the PSQI, was longer in the REM-OSA group than in the NREM-OSA group (*p* = 0.007). The sleep duration and sleep efficiency of the REM-OSA group was lower than that of the NREM-OSA group (*p* = 0.047, *p* = 0.001). These differences were statistically significant.

**Table 4:** Comparison of excessive sleepiness and sleep quality among the study participants.

Variables	NREM-OSA	REM-OSA	t/z	P
ESS score	8.000 (3.000–12.000)	8.000 (3.000–14.000)	−0.516	0.607
PSQI score	8.52 ± 4.244	10.18 ± 4.324	−2.901	0.004
Subjective sleep quality	1.00 (1.00–2.00)	1.00 (1.00–2.00)	−1.015	0.312
Sleep latency	1.00 (1.00–3.00)	3.00 (1.00–3.00)	−4.227	0.000
Seep duration	1.00 (1.00–2.00)	2.00 (1.00–3.00)	−1.993	0.047
Habitual sleep efficiency	1.00 (1.00–3.00)	2.00 (1.00–3.00)	−3.241	0.001
Sleep disturbances	1.30 ± 0.493	1.30 ± 0.490	0.058	0.954
Use of sleeping medication	0.00 (0.00–0.00)	0.00 (0.00–0.00)	0.395	0.693
Daytime dysfunction	2.00 (0.00–3.00)	2.00 (0.00–3.00)	−0.311	0.756

ESS, Epworth sleepiness scale; PSQI, Pittsburgh Sleep Quality Index.

### 3.5 Logistic regression of REM-OSA

As shown in Table 5, WBC, hs-CRP, Basal ganglia, PSQI, AHI and MRS were identified as influencing factors for REM-OSA patients in the cerebral infarction cohort according to the unadjusted logistic regression analysis. The adjusted model showed that hs-CRP, basal ganglia infarction, AHI and MRS were factors that significantly increased the risk of REM-OSA.

**Table 5:** Unadjusted and adjusted ORs (95% CIs) for variables associated with REM-OSA patients.

Variables	B	OR	95% (CI)	P
Unadjusted				
Age, years	−0.003	0.997	0.973, 1.021	0.793
Female sex	−0.626	0.535	0.312, 0.916	0.023
BMI	0.027	1.028	0.954, 1.106	0.470
Diabetes mellitus, %	−0.093	0.912	0.530,1.566	0.737
History of coronary heart disease, %	0.118	1.125	0.603,2.098	0.711
WBC, 10^9^/L	0.198	1.219	1.080, 1.375	0.001
NE, 10^9^/L	0.093	1.098	0.986, 1.222	0.088
hs-CRP, mg/L	0.185	1.203	1.114,1.299	0.000
Basal ganglia, %	−0.983	0.374	0.191,0.733	0.004
PSQI	0.089	1.093	1.028, 1.162	0.005
AHI	−0.039	0.961	0.943, 0.981	0.000
MRS	0.504	1.656	1.140, 2.405	0.000
Adjust				
WBC, 10^9^/L	0.123	1.131	0.993,1.289	0.064
hs-CRP, mg/L	0.163	1.177	1.086,1.275	0.000
Basal ganglia, %	0.858	2.359	1.165,4.776	0.017
PSQI	0.065	1.067	0.997,1.142	0.060
AHI	−0.051	0.951	0.929,0.973	0.000
MRS	0.434	1.543	1.037,2.296	0.032

BMI, body mass index; WBC, white blood cell; NE, neutrophilic granulocyte; hs-CRP, high-sensitivity C-reactive protein; PSQI, Pittsburgh Sleep Quality Index; AHI, Apnea Hypopnea Index; MRS, Modified Rankin Scale.

### 3.6 Pearson correlation analysis among age, WBC, NE, hs-CRP, basal ganglia, PSQI, MRS, and AHI_REM_

**Figure 3:**
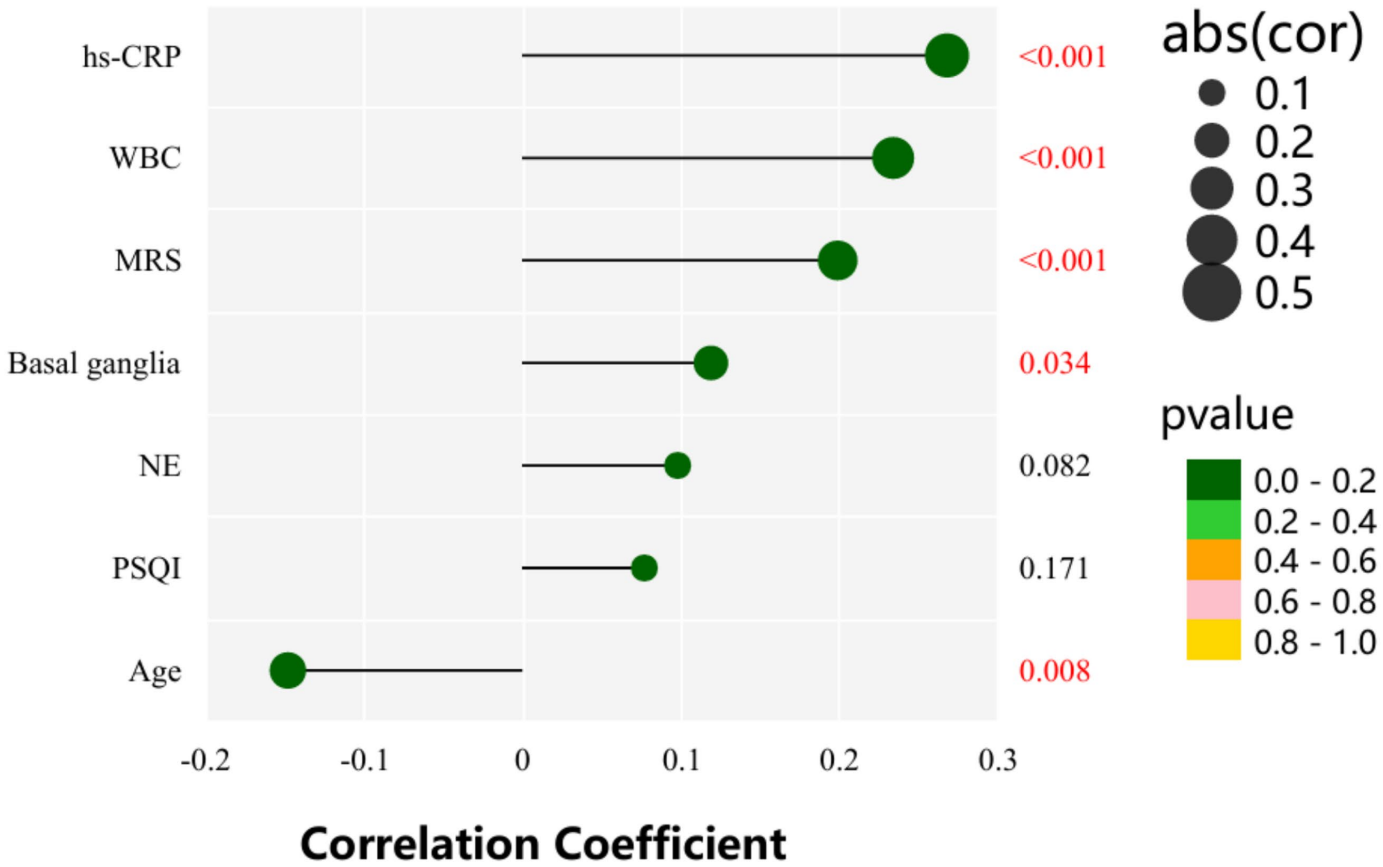
Pearson correlation between variables and REM-AHI.

As shown in Table 6 and Figure 3, AHI_REM_ was significantly positively correlated with Age, WBC, hs-CRP, Basal ganglia and MRS (*r* = −0.148, *p* = 0.008; *r* = 0.234, *p* = 0.000; *r* = 0.268, *p* = 0.000; *r* = 0.119, *p* = 0.034; *r* = 0.199, *p* = 0.000).

**Table 6:** Pearson correlation analysis between variables and AHI_REM_.

Variables	r	*p*
Age, years	−0.148	0.008
WBC, 10^9^/L	0.234	0.000
NE,10^9^/L	0.098	0.082
hs-CRP, mg/L	0.268	0.000
Basal ganglia, %	0.119	0.034
PSQI	0.077	0.171
MRS	0.199	0.000

### 3.7 Comparison of sleep monitoring results between two groups of patients with cerebral infarction

As shown in Table 7, the number of NREM apnea events, AHIN_REM_, AHI, Nadir SpO_2_, ODI and respiratory event-related arousal index in the REM-OSA group were lower than those in the NREM-OSA group. Additionally, the AHI_REM_ and longest apnea time in the REM-OSA group was greater than that in the NREM-OSA group, and this difference was statistically significant (*p* < 0.05).

**Table 7:** Polysomnographic findings of the study population.

Variables	NREM-OSA	REM-OSA	t	*p*
Sleep time, min	365.206 ± 123.854	369.437 ± 123.575	−0.254	0.800
Sleep latency, min	7.300 (3.200–15.000)	9.100 (3.900–26.500)	−1.954	0.054
Sleep efficiency	80.100 (67.300–88.100)	76.400 (69.100–85.500)	1.110	0.268
REM sleep, % of TST	17.500 (11.000–25.200)	18.800 (11.600–25.700)	−0.528	0.598
REM latency, min	66.000 (38.000–129.000)	68.500 (30.000–118.500)	−0.039	0.969
REM sleep, min	63.000 (36.500–95.500)	70.500 (37.000–105.000)	−0.513	0.609
N1 sleep, min	25.500 (11.900–46.500)	18.800 (9.500–43.500)	1.149	0.252
N2 sleep, min	211.245 ± 89.764	195.454 ± 93.221	1.295	0.196
N3 sleep, min	34.000 (12.500–68.200)	46.000 (17.000–84.500)	−2.558	0.011
Number of sleep apnea, REM	4.000 (0.000–14.000)	9.000 (5.000–19.000)	−1.271	0.205
AHI_REM_, events/h	10.900 (6.500–21.300)	4.400 (1.000–12.200)	−4.067	0.000
Number of sleep apnea, NREM	29.000 (11.000–69.000)	9.000 (4.000–22.000)	0.784	0.000
AHI_NREM_, events/h	7.300 (3.000–15.800)	2.500 (1.200–6.900)	7.557	0.000
AHI, events/h	25.900 (14.500–41.700)	18.200 (12.500–25.600)	5.631	0.000
AHI after CPAP, events/h	3.603 ± 1.770	3.500 (2.500–4.900)	−0.748	0.455
Longest hypoventilation time, s	93.896 ± 29.543	95.293 ± 26.267	−0.360	0.719
Longest apnea time, s	75.989 ± 22.835	51.000 (33.000–83.000)	−5.069	0.000
Mean SpO_2_, %	94.510 ± 2.149	94.669 ± 2.154	−0.550	0.583
Nadir SpO_2_, %	84.310 ± 6.185	81.620 ± 5.032	3.764	0.000
ODI, events/h	24.100 (12.600–41.800)	15.600 (9.300–26.900)	4.692	0.000
Arousal index, events/h	28.700 (17.900–39.200)	25.200 (14.100–34.200)	1.591	0.192
Respiratory event-related arousal index, events/h	3.100 (1.600–5.300)	5.400 (2.100–10.900)	−5.060	0.000

REM, Rapid Eye Movement; AHI, apnea Hypopnea Index; PAP, positive airway pressure; ODI, Oxygen Desaturation Index.

## 4 Discussion

REM-OSA may be accompanied by increased excitability of the sympathetic nervous system and instability of the circulatory system (19), which may be associated with the prognosis of cerebral infarction. However, the relationship between REM-OSA and the prognosis of cerebral infarction has rarely been studied. In this study, REM-OSA and NREM-OSA were compared among patients who suffered a cerebral infarction, and our main finding was that, compared with patients with NREM-OSA, REM-OSA was associated with higher mRS scores and poorer prognosis.

This study revealed that REM-OSA group had higher MRS scores and a worse prognosis (*p* = 0.024). Studies have shown that the number of synapses created increases with the duration of REM sleep and that REM sleep provides the optimal conditions for the activation of sources necessary for the maturation of the central nervous system (20). Patients with REM-OSA who suffer a cerebral infarction sleep for shorter periods and experience poor sleep quality, thus leading to a reduction in the duration of REM sleep and therefore fewer new synapses, thus hindering the maturation of the central nervous system. In addition, several studies have indicated that regional brain tissue ischemia in patients with cerebral infarction may lead to an increased frequency of respiratory events during REM sleep. The underlying mechanisms can be summarized as follows: (1) Regional brain tissue ischemia may impair the brainstem and hypothalamus, which regulate respiration and the autonomic nervous system. Neurological dysfunction resulting from ischemia could disrupt normal respiratory regulation, thereby increasing the incidence of apnea and hypopnea events (21). (2) Alterations in sleep architecture: Patients with cerebral infarction frequently experience sleep architecture disorders. Physiological changes during REM sleep, such as reduced muscle tone and increased airway collapsibility, may exacerbate respiratory events (22). (3) Intermittent hypoxia and arousal responses: Localized brain tissue ischemia may induce nocturnal intermittent hypoxia, triggering arousal responses that disrupt normal sleep-breathing patterns and increase the frequency of respiratory events (23). Beyond the effects of nocturnal intermittent hypoxia and sleep fragmentation caused by OSA, metabolic activity is elevated in certain brain regions during REM sleep (e.g., the medial prefrontal cortex and insular cortex), which are particularly vulnerable to hypoxia and hypoperfusion. Respiratory events may cause damage to these regions, potentially leading to daytime hypoperfusion (24). In conclusion, REM-OSA may be closely associated with the poor prognosis of cerebral infarction.

In addition, some studies have shown that local brain tissue ischemia in patients who suffer a cerebral infarction may lead to an increase in the number of respiratory events experienced during REM sleep, and apnoea and hypopnea events during REM sleep can lead to daytime hypoperfusion in the ventromedial prefrontal lobe and frontal insular lobe (24); therefore, the prognosis of a cerebral infarction is adversely affected.

The findings of this study demonstrated that white blood cell count (WBC), neutrophils (NE), and high-sensitivity C-reactive protein (hs-CRP) levels were significantly elevated in the REM-OSA group compared to the NREM-OSA group (*p* < 0.05). Furthermore, WBC and hs-CRP exhibited a positive correlation with AHI_REM_ (*r* = 0.234, *p* = 0.000; *r* = 0.268, *p* = 0.000). Logistic regression analysis corroborated that increased WBC and hs-CRP levels serve as independent risk factors for REM-OSA. This observation may be attributed to the activation of the sympathetic nervous system, heightened cerebral metabolic demand, and intermittent hypoxia during REM sleep, which collectively stimulate the NF-κB pathway and trigger the release of inflammatory mediators (3, 25). Notably, despite a lower total AHI, REM-OSA patients exhibited an independent increase in hs-CRP levels (OR = 1.177, *p* = 0.000), suggesting that REM-OSA may exacerbate tissue damage via a distinct hypoxia-inflammatory mechanism. In contrast, the suppression of the sympathetic nervous system and respiratory stabilization during NREM sleep contribute to maintaining lower levels of inflammation (26, 27). These results underscore the necessity of targeted inflammation monitoring and optimized CPAP therapy for patients with REM-OSA.

The present study revealed that, in patients with cerebral infarction, the subjective sleep quality of the REM-OSA group was inferior to that of the NREM-OSA group (*p* = 0.004). However, no statistically significant difference was observed in the degree of sleepiness between the two groups. The impaired sleep quality in REM-OSA patients was characterized by difficulties in initiating sleep, reduced sleep duration, and diminished sleep efficiency. This phenomenon may be attributed to the significantly heightened sympathetic nervous activity, cardiovascular instability (28), and concentrated respiratory events and micro-arousals during REM sleep, which result in sleep fragmentation and consequently affect overall sleep quality.

The results showed that the proportion of patients with basal ganglia infarction in the REM-OSA group was significantly higher than in the NREM-OSA group (83.1% vs. 64.8%, *p* = 0.003). Basal ganglia infarction was also identified as an independent risk factor for REM-OSA (OR = 2.359, *p* = 0.017). The basal ganglia have unique physiological functions during REM sleep, including the regulation of movement and mood (29). The findings of this study may be related to the unique pathophysiological mechanisms that occur during REM sleep. Firstly, intermittent hypoxia caused by respiratory events during REM sleep can selectively damage the microcirculation in the basal ganglia, which are highly sensitive to hypoxia (30). Secondly, sympathetic hyperactivation during REM sleep may exacerbate basal ganglia ischaemia through blood pressure fluctuations and vasoconstriction (19). These factors may act together to increase the vulnerability of the basal ganglia (31). Additionally, the basal ganglia are involved in the regulation of REM sleep (29), forming a vicious circle of ‘hypoxia-sympathetic activation-basal ganglia injury-REM disorder’. While previous studies have disputed the association between OSA and infarct location, this study suggests that REM-OSA may specifically affect the basal ganglia (32–34). This needs to be verified in future studies using multimodal imaging and dynamic PSG monitoring.

In this study, we found that the total Apnea-Hypopnea Index (AHI) was significantly lower in the REM-OSA group compared to the NREM-OSA group (*p* = 0.000), but the AHI_REM_ was significantly higher (*p* = 0.000). By incorporating AHI as a covariate in a binary logistic regression model, we determined that REM-OSA was still significantly associated with a poor prognosis of cerebral infarction, even after adjusting for total AHI (refer to Table 5). REM-OSA patients experienced a higher concentration of respiratory events during REM sleep, with longer event durations and more significant decreases in oxygen saturation. This indicates that the poor prognosis in patients with cerebral infarction is strongly linked to REM-phase-specific breathing disorder patterns, rather than just the overall severity of sleep apnea. During REM sleep, there is typically a higher arousal threshold due to increased cholinergic inhibition and reduced noradrenergic activity in the brainstem, which suppresses cortical arousal responses to respiratory stimuli (35). However, in REM-OSA patients, recurrent respiratory events during REM sleep lead to cumulative hypoxic stress and exaggerated chemoreflex sensitivity. This chronic intermittent hypoxia triggers neuroplastic changes in the brainstem, resulting in dysregulation of the arousal circuitry (36). Consequently, the physiological inhibitory mechanisms are overridden, leading to a paradoxical decrease in the arousal threshold despite REM-specific neural suppression. Additionally, white blood cell count (WBC) and hypersensitive C-reactive protein (hs-CRP) were significantly higher in the REM-OSA group than in the NREM-OSA group in the present study (*p* < 0.05), and these inflammatory factors may affect neuromodulation in the brain, thereby lowering the arousal threshold. The above mechanisms explain why the REM-OSA group in the present study exhibited more frequent microarousals (longer sleep latency and lower sleep efficiency, as shown in Table 4), which in turn exacerbated sleep fragmentation and hindered neural repair after cerebral infarction. The study also found significantly lower minimum oxygen saturation in the REM-OSA group (81.62% vs. 84.31%, *p* < 0.001) with a longer duration of the longest apnea. This is consistent with the literature: respiratory events during REM typically last 16.7% ~ 29.8% longer than those during NREM, and the reduction in blood oxygen saturation increases by 36.1% ~ 48.0% (37). This further suggests that hypoxia is more severe during REM sleep in REM-OSA patients. Therefore, the assessment of REM-OSA severity based on AHI alone has limitations, and special attention needs to be paid to the degree of REM-phase-specific hypoxia, timely control of REM-OSA, and individualized diagnostic and therapeutic protocols to improve the prognosis of patients with cerebral infarction.

This study found that age was negatively correlated with the severity of REM-OSA (AHI_REM_ and age correlation coefficient r = −0.154, *p* = 0.020), which was consistent with previous studies indicating that REM-OSA may be more prominent in younger populations (38). The underlying mechanism may be related to age-related changes in sleep architecture. The decreased duration and stability of REM sleep in elderly patients may lead to weakened performance of REM-specific respiratory events. However, young people’s sympathetic nervous system is more sensitive to the transition between sleep stages (39), therefore, younger patients exhibit higher sympathetic nerve activity during REM sleep, which may exacerbate respiratory disorders. Therefore, in clinical practice, we should pay special attention to young REM-OSA patients. Early detection and treatment of this disease are essential. In the future, larger samples and longitudinal studies are needed to further clarify the interaction between age, REM sleep pathophysiology, and the OSA phenotype, particularly regarding stroke prognosis.

To improve the prognosis of cerebral infarction, active treatment of REM-OSA is important. The total AHI of patients with REM-OSA is generally low, predominantly suggesting a mild to moderate case. Whether treatment is needed is debated. Continuous positive airway pressure (CPAP) is the first-line treatment for OSA, with a recommended usage duration of 4 h (40). However, given that REM-OSA mainly occurs in the latter half of sleep, it is challenging for 4 h of CPAP to cover all the apnoea events in the REM sleep phase. Some studies revealed that 7 h of CPAP therapy can affect more than 80% of REM sleep (41), but this needs to be confirmed through studies with larger sample sizes. Additionally, poor patient compliance further contributes to the low cure rate of REM-OSA, ultimately impacting the clinical outcome of patients who suffer a cerebral infarction. Given the potential influence of CPAP adherence on stroke recovery, the lack of this information limits our ability to fully interpret the treatment implications of our findings. Future studies should include detailed CPAP usage data to better understand its impact on the prognosis of cerebral infarction patients with REM-OSA and NREM-OSA.

### 4.1 Limitations

First, the sample sizes of the REM-OSA and NREM-OSA groups were unequal. Second, patients underwent only one polysomnography, potentially overlooking night-to-night variability. Third, the single-center design in China may limit generalizability to other populations. Fourth, stroke subtypes (e.g., large-artery atherosclerosis, cardioembolism) were not classified. This omission may influence the distribution of OSA phenotypes and associated outcomes, as different stroke etiologies could exhibit distinct pathophysiological interactions with sleep-disordered breathing (42, 43). Fifth, while inflammatory indices (WBC, hs-CRP) were analyzed, key biomarkers such as IL-6 and TNF-*α* were not assessed. Future studies should incorporate these mediators to elucidate the precise role of inflammation in REM-OSA-related neural injury.

## 5 Conclusion

The study revealed that REM-OSA is associated with poorer neurological function recovery and worse clinical outcomes in cerebral infarction patients compared to NREM-OSA. These findings suggest that early diagnosis and management of REM-OSA may contribute to improving prognosis of cerebral infarction.

## Data Availability

The original contributions presented in the study are included in the article/supplementary material, further inquiries can be directed to the corresponding authors.

## Glossary

OSA: obstructive sleep apnea
REM-OSA: rapid eye movement related obstructive sleep apnea
PSG: polysomnography
ESS: Epworth Sleepiness Scale
PSQI: Pittsburgh Sleep Quality Index
NREM-OSA: non-rapid eye movement related obstructive sleep apnea
BMI: body mass index
WBC: white blood cell
NE: neutrophilic granulocyte
hs-CRP: high-sensitivity C-reactive protein
TG: triglyceride
TC: total cholesterol
LDL-C: low density lipoprotein cholesterol
Hcy: homocysteine
SCR: serum creatinine
HbA1c: glycosylated haemoglobin
AHI: apnea-hypopnea index
ODI: oxygen desaturation index
CI: confidence interval
NHISS: National Institute of Health Stroke Scale
mRS: Modified Rankin Scale
